# Insufflation of Salt in the Treatment of Neuralgia

**Published:** 1890-03

**Authors:** 


Insufflation of Salt in the Treatment of Neuralgia.—At a recent meeting of the Edinburgh Medico-Chirurgical Society, Dr. George Leslie gave the details of thirty or forty cases of facial and other neuralgias, cephalalgia, odontalgia, etc., which had been cured, in most instances instantaneously, by insufflation of powdered common salt through the anterior nares. The salt was either "snuffed" or blown up the nostrils. He had been unsuccessful in only two cases; both of these were cases of old standing, which had been treated frequently by morphine injections. In one of them excision of the nerve had been practised.—*Cincinnati Lancet-Clinic*.

THE BACILLUS OF WARTS.—Dr. Kunemann has found in sections of warts (*verruca vulgaris*) a bacillus which is always present in the prickle layer. It has distinctive qualities as regards its capacity for color, and is found both between and in the cells. Its form is that of exceedingly delicate slender rods, the thickness bearing the proportion to the length of one to six. It is seldom found in the skin surrounding the warts, but is found most plentiful when the wart is recent.—*Medical Age.*

G. W. WATSON, in the *Ohio Journal of Dental Science*, says: "I have very good authority for saying that diseased roots and teeth have a great deal to do in starting tubercular trouble in the lymphatic glands of people predisposed to this disease. Tubercle bacilli, gaining admission to the jaw through the diseased teeth, speedily infect the structures in their neighborhood. It would be right, therefore, for us to look well to the teeth of patients having a tubercular tendency, and see that they keep their mouth in a thoroughly healthy and aseptic condition.

SALICYLIC ACID IN THE TREATMENT OF CORNS.—Moisten the corn with a solution of salicylic acid, and sprinkle over the surface a layer of pure acid. Cover with a small pad of salicylated cotton and oilsilk. This dressing should be renewed every four or five days, until the corn is desiccated, when it is easily removed. One or two weeks' treatment is usually sufficient.—*Revue Générale de Clinique et de Thérapeutique.*

FOR A case of phthisis at the clinic, Prof. Da Costa directed ol. morrhuæ f℥iv t. d.; inhalations of terebene ʒj to Oj boiling water, and the following prescription:

℞—Liquor. potassii arsenitis . . . . . . . . . . . . . . . . m. iij.
Tinct. nucis vomicæ . . . . . . . . . . . . . . . . . . . gtt. v.
Tinct. cinchonæ comp . . . . . . . . . . . . . . . . . . f℥ j.
M. Sig—*ter die.* —*Canada Med. Record.*

GONORRHEA.

The number of different injections for this trouble is legion, but Garretson says he has always found this one satisfactory:

℞—Chloral hydrat.
Zinci sulphatis . . . . . . . . . . . . . . . . . . . . . āā gr. ij.
Aquæ . . . . . . . . . . . . . . . . . . . . . . . . . . ℥ j.
M. —*Med. Summary.*

VACCINATION ON THE LEG.—A French practitioner, in the course of a large number of revaccinations, was struck with the fact that the operation was far more successful when performed on the leg than when the arm was selected. Among 177 cases, the percentage of failures was 45.45 on the leg, as compared with 53.84 on the arm.—*Med. Press and Circular.*

### TREATMENT OF PRURITUS ANAL AND VULVÆ.

| | |
|---|---|
| R—Hyposulphite of soda | 15.0 |
| Acid carbonic | 2.5 |
| Glycerini | 8.0 |
| Aquæ | 120.0 |

M—Sig—Lotio.—*Dublin Journal Med. Sciences.*

### CORYZA SNUFF.

| | |
|---|---|
| R—Morphiæ muriat | gr. ii. |
| Bismuth. subnitr | zi. |
| Pulv. accaciæ | zii. |

M. Fiat pulv.

(*Canada Med. Record.*)

### TO ARREST VOMITING DURING PREGNANCY.

| | |
|---|---|
| R—Cerii oxalat. | |
| Ipecacuanhæ | aa gr. j. |
| Creasoti | gtt. ij. |

M. Sig—To be taken every hour.—*Med. Summary.*

### COUGH MIXTURE.

| | |
|---|---|
| Cod liver oil | ℥ ij. |
| Honey | ℥ ij. |
| Lemon juice | ℥ ij. |

One to two teaspoonfuls three times a day.—*Med. Summary.*

It is a common occurrence for children to get beans, grains of corn, and other foreign substances up their nose. This simple remedy is worth remembering: Get the child to open its mouth, apply your mouth over it, and blow hard. The offending substance will be expelled from its mouth.—*Medical Classics.*

Poisonous Thread.—The *Sanitary News* draws attention to the fact that silk thread is soaked in acetate of lead to increase its weight, and persons who pass it through the mouth in threading needles, and then bite off the thread with the teeth, have suffered from lead poisoning.

Flint says: "I have never known a dyspeptic to recover vigorous health who undertook to live after a strictly regulated diet, and I have never known an instance of a healthy person living according to a strict dietetic system who did not become a dyspeptic."

The French method of administering castor oil to children, is to pour the oil into a pan over a moderate fire, break an egg into it, and stir it up; when it is done, flavor with a little salt or sugar or currant jelly.

PROF. BARTHOLOW recommends for habitual constipation a few minims of wine of tobacco, taken at bedtime. It acts by increasing the secretion and causing peristaltic action.—*College and Clinical Record.*

FOR ingrowing toe nails, use equal parts of mutton tallow, castile soap, and white sugar, made into a salve. Apply until the swelling is down, then trim the nail in the center.

TO RENDER MILK MORE DIGESTIBLE.—When milk agrees badly with patients, it is said that shaking it thoroughly, just as lemonade is shaken, will render it more digestible·—*Ex.*

M. GALLE has observed in persons who use the telephone much, symptoms of "aural over-pressure," caused by the condition of almost constant strain of the auditory apparatus.

NOTHING so quickly restores tone to exhausted nerves and strength to a weary body, as a bath containing an ounce of aqua ammonia to each pail of water.—*Annals of Hygiene.*

FOR BED SORES, Prof. Rosenbach recommends that lanoline be rubbed into a red spot denoting an impending bed sore. He has had unvarying success with it.—*Ex.*

EVERY one should have eight hours' sleep, and pale, thin, nervous persons require ten, which should be taken regularly, in a well ventilated room.

---

"DOCTOR, if a plain young man, named Blinker, calls on you to-day for a prescription, don't let him have it."

"Why not?"

"He wants something to restore his appetite, and he boards at my house."

---

"AND, DOCTOR, can you make this bloom again?" asked Father Time, pointing to a specimen of the vintage of 1840. "I was once a footlight favorite, and men showered me with pearls and diamonds. O, can I be young again?" she exclaimed with the fervor of a maiden of sixty-two summers. "You will be Queen of the May," responded Dr. Brown-Sêquard, as he proceeded to his laboratory and slaughtered a fresh Guinea-pig.

